# Genome Sequence of *Jaltomata* Addresses Rapid Reproductive Trait Evolution and Enhances Comparative Genomics in the Hyper-Diverse Solanaceae

**DOI:** 10.1093/gbe/evy274

**Published:** 2019-01-04

**Authors:** Meng Wu, Jamie L Kostyun, Leonie C Moyle

**Affiliations:** 1 Department of Biology, Indiana University Bloomington; 2 Department of Plant Biology, University of Vermont

**Keywords:** comparative genomics, de novo assembly, floral evolution, potato, tomato, transposable element

## Abstract

Within the economically important plant family Solanaceae, *Jaltomata* is a rapidly evolving genus that has extensive diversity in flower size and shape, as well as fruit and nectar color, among its ∼80 species. Here, we report the whole-genome sequencing, assembly, and annotation, of one representative species (*Jaltomata sinuosa*) from this genus. Combining PacBio long reads (25×) and Illumina short reads (148×) achieved an assembly of ∼1.45 Gb, spanning ∼96% of the estimated genome. Ninety-six percent of curated single-copy orthologs in plants were detected in the assembly, supporting a high level of completeness of the genome. Similar to other Solanaceous species, repetitive elements made up a large fraction (∼80%) of the genome, with the most recently active element, *Gypsy*, expanding across the genome in the last 1–2 Myr. Computational gene prediction, in conjunction with a merged transcriptome data set from 11 tissues, identified 34,725 protein-coding genes. Comparative phylogenetic analyses with six other sequenced Solanaceae species determined that *Jaltomata* is most likely sister to *Solanum*, although a large fraction of gene trees supported a conflicting bipartition consistent with substantial introgression between *Jaltomata* and *Capsicum* after these species split. We also identified gene family dynamics specific to *Jaltomata*, including expansion of gene families potentially involved in novel reproductive trait development, and loss of gene families that accompanied the loss of self-incompatibility. This high-quality genome will facilitate studies of phenotypic diversification in this rapidly radiating group and provide a new point of comparison for broader analyses of genomic evolution across the Solanaceae.

## Introduction

Understanding the genetic substrate of trait diversification is a longstanding goal in evolutionary biology. Diversification can involve a range of genetic changes, including point mutations in coding or regulatory regions, or structural variation such as chromosomal inversions or gene duplications (Stapley et al. 2010; Berner and Salzburger 2015). Until recently, these data have been challenging to generate for all but the best developed model species. However, the emergence of next-generation and single molecule sequencing technologies now allows the rapid generation of diverse genomic resources, including whole-genome sequences, transcriptome sequences, and genome-wide marker panels for a much broader range of taxa (Stapley et al. 2010; Ellegren 2014; Bleidorn 2016). Comparative genomic analyses of related organisms provide opportunities to quantify species differences in genome size, complexity, noncoding features, and molecular evolution within genic regions, as well as structural differences in the number and identity of members of specific gene families, or in classes of transposable elements (TEs). In combination with data on specific phenotypic and functional trait variation, comparative genomic analyses can also evaluate the role of different genomic changes in both general patterns of lineage diversification and lineage-specific adaptive evolution. In addition to deciphering mechanisms of genome evolution, these data can be used to address the genetics of phenotypic diversity associated with adaptation and speciation, across groups of closely related, ecological diverse, species (Brawand et al. 2014; Zhang et al. 2014; Novikova et al. 2016; Yin et al. 2018).

The plant genus *Jaltomata* is one such rapidly radiating clade, consisting of 60–80 species estimated to have arisen within the last 5 Myr or less (Mione 1992; Miller et al. 2011; Wu et al. 2018). *Jaltomata* is closely related to both *Solanum* (which includes tomato, potato, and eggplant) and *Capsicum* (peppers), and together these three form a clade that is sister to the rest of the Solanaceae, a highly diverse plant family that also contains other economically important genera such as *Nicotiana* (tobacco) and *Petunia* (Bohs and Olmstead 1997; Olmstead et al. 1999; Walsh and Hoot 2001; Olmstead et al. 2008; Särkinen et al. 2013). Because multiple Solanaceous species already have whole-genome sequences with gene annotations, an additional high-quality genome in the key phylogenetic position held by *Jaltomata* provides a valuable resource for clarifying the evolutionary relationships among these important clades, and for comparative analyses of genomic, genetic, and phenotypic evolution across the family. For example, although most Solanaceae species have the same base number of chromosomes (*X* = 12; *Petunia* is the exception, with *X* = 7), estimated genome sizes vary drastically (e.g., 4-fold genome-size difference between tomato and hot pepper) (Kim et al. 2014; Xu et al. 2017) suggesting that repetitive element divergence might be a key contributor to genome-size variation. Further, comparative analyses of gene family evolution and sequence divergence could identify changes associated with important ecological trait variation (e.g., identification of tandemly duplicated genes involved in the capsaicin biosynthesis pathway specifically in hot pepper [Kim et al. 2014; Qin et al. 2014]). Comparative genomic analysis including *Jaltomata* therefore could reveal both large- and small-scale genetic changes responsible for genomic, phenotypic, and functional differentiation among these important clades.

Apart from its key position within the Solanaceae, the genus *Jaltomata* itself varies widely in ecological range, vegetative characters, physiology, and reproductive form and function (Haak et al. 2014), making it a valuable emerging model for studies of adaptive diversification and evolution of novel traits. Like its close relatives in *Solanum* and *Capsicum*, *Jaltomata* has its highest diversity in the Andean region of South America, although some species’ ranges extend into Central America and the southwestern United States. Its species occur in a broad range of habitats, including tropical forests, coastal lowlands, and lomas (discrete fog-misted communities surrounded by arid desert) (Mione 1992; Mione and Yacher 2005). Unlike its close relatives, *Jaltomata* lineages exhibit a striking and unique diversity of derived floral traits, especially in corolla (petal) shapes—that include rotate, campanulate (bell-shaped), and tubular forms (Miller et al. 2011; Kostyun and Moyle 2017)—and in the amount and color of nectar produced, which ranges from small amounts of nectar that is essentially colorless to copious amounts of deep red nectar (Hansen et al. 2007). In comparison, close relatives *Solanum* and *Capsicum* predominantly have flatter rotate corollas, and either colorless to pale yellow nectar (*Capsicum*) or no floral nectar at all (*Solanum*) (Knapp et al. 2004). *Jaltomata* species also vary in mature fruit color, including species that have either purple, red, orange, or green fruit at maturity; this fruit color variation appears to characterize three major subclades within the genus as separate dark purple-, red-, and orange-fruited clades (Miller et al. 2011; Särkinen et al. 2013; Wu et al. 2018). The orange-fruited clade (comprising ∼50 species) is the subgroup containing most novel derived floral trait variation and is estimated to have diverged within the last 1.5 Myr, consistent with a very rapid recent radiation of floral and reproductive diversity that likely drew upon multiple sources of genetic variation (Wu et al. 2018). *Jaltomata* is also distinctive among Solanaceae genera in that all examined species are self-compatible (SC) (Mione 1992) (Kostyun JL and Mione T, unpublished data), whereas most other genera—including *Solanum* and *Capsicum*—exhibit genetically determined self-incompatibility (SI) in some or all species (Goldberg et al. 2010). The availability of a high-quality genome in this genus could thus help to assess genome features specific to *Jaltomata* to identify genetic changes that might accompany or drive its unique and rapid trait evolution.

In this study, we generated a high-coverage and almost complete genome of one *Jaltomata* species, *J. sinuosa*, by adopting a hybrid assembly strategy using PacBio long reads and Illumina short reads. Using this newly assembly genome, we performed comparative genomic analyses with six additional high-quality genomes in the Solanaceae. We found that different topologies of *Jaltomata*, *Solanum*, and *Capsicum* were supported by a large number of individual gene trees, suggesting a complex history of rapid divergence and hybridization in the common ancestors of these three genera. Within *Jaltomata*, we identified a recent expansion of *Gypsy* elements, a superfamily of long terminal repeat-retrotransposons (LTR-RTs), around 1–2 Ma that likely contributed to the genome-size expansion of *Jaltomata*. In addition, assessing genome features specific to *Jaltomata* identified genetic changes that could have contributed to rapid trait evolution in the genus, including loci with lineage-specific patterns of adaptive evolution, the loss of gene families that accompanied the loss of self-incompatibility, and the expansion of gene families potentially involved in novel trait development in *Jaltomata*. We discuss the significance of this genome assembly in the light of phenotypic diversity in this rapidly radiating group and genomic evolution across the economically important plant family Solanaceae.

## Materials and Methods

### Species Selection and Tissue Sampling

Among the 60–80 species within the genus *Jaltomata*, *J.sinuosa* was chosen for whole-genome sequencing because estimated heterozygosity within this species is the lowest of all evaluated species (Wu et al. 2018 and this article), potentially facilitating genome assembly. Further, although *J. sinuosa* itself has rotate corollas with light-yellow nectar (i.e., similar to ancestral trait states in the genus; Miller et al. 2011; Kostyun et al. 2017), it belongs within the recently diverged (<1.5 Myr) and highly diverse (∼50 species) orange-fruited clade, which incorporates the majority of novel floral diversity within the genus (Miller et al. 2011; Wu et al. 2018). To obtain genomic DNA, young leaf tissue was collected from a single individual of *J. sinuosa* grown in the Indiana University greenhouse (voucher available at IND herbarium), and flash frozen with liquid nitrogen. DNA was extracted using Qiagen DNeasy Plant Kits, purified with ethanol precipitation, and quality checked using Nanodrop and gel electrophoresis. Approximately 60-μg genomic DNA was provided to the Duke University Sequencing and Genome Technologies facility for library preparation and sequencing: 4 large-insert (15–20 kb) libraries were constructed and sequenced in 67 SMRT cells on the Pacific Biosciences (PacBio) platform.

### Genome Assembly

We adopted a hybrid assembly approach (Koren et al. 2012) in which relatively high-accuracy Illumina paired-end reads (148×) were used to trim and correct low base-call-accuracy PacBio long reads (25×). Initially, we used two different genome-assembly strategies DBG2OLC (Ye et al. 2016) and MaSuRCA v3.2.2 (Zimin et al. 2017), and then evaluated the completeness of genome assembly for each using 1,515 plant near-universal single-copy ortholog within BUSCO v3 (Simão et al. 2015). We found the assembly output from MaSuRCA was much better than that from DBG2OLC, in terms of assembly coverage, contig N50, and genome completeness (supplementary table S1, Supplementary Material online). Thus, the initial assembly from MaSuRCA was used for all further analyses. Genome size was also estimated within the MaSuRCA pipeline based on the *k*-mer abundance distribution.

To remove potential contaminants in our assembly, all scaffolds were aligned against the NCBI nonredundant nucleotide sequence database using BlastN with a cutoff of E-5. Scaffolds were assigned to the closest reference based on the best combined hit score, and any scaffolds assigned to a nonplant species were removed (Bolger et al. 2014). In total, 22 small scaffolds were filtered out, consisting of 240,347 bases, leaving 7,667 scaffolds in the genome assembly. To estimate assembly accuracy at the nucleotide level, we estimated the discrepancy between the long-read sequences used in the initial genome assembly and the high-quality Illumina reads, the latter of which were used to error correct the long-read base calls (see supplementary text, Supplementary Material online).

### Repeat Annotation

We followed the “Repeat Library Construction-Advanced” steps from the MAKER-P pipeline (Campbell et al. 2014) to generate a *Jaltomata*-specific repeat library. Briefly, miniature inverted TEs (MITEs) were detected by MITE-Hunter (Han and Wessler 2010); LTRs were constructed using LTRharvest (Ellinghaus et al. 2008) followed by LTR_retriever (Ou and Jiang 2018); and other repetitive sequences were identified using RepeatModeler (http://www.repeatmasker.org/RepeatModeler.html; last accessed January 9, 2019). RepeatMasker (http://www.repeatmasker.org) was then used to mask repeat elements in the assembled genome by searching for all homologous repeats in the species-specific library. To compare the recent activity of LTR-RTs, we also applied the same approach (i.e., LTRharvest followed by LTR_retriever) to annotate the full-length LTR-RTs in four other genomes from *Solanum* and *Capsicum*, including *S. lycopersicum*, *S. tuberosum*, *S. pennellii*, and *C. annuum* (see supplementary text, Supplementary Material online). The timing of LTR-RT bursts was based on the distribution of times inferred for each full-length element based on sequence divergence between its direct repeats, in LTR_retriever (Ou and Jiang 2018). Briefly, insertion time (*T*) of each full-length LTR-RT was estimated as *T* = *K*/2*μ*, where *K* is the divergence rate between the two terminal repeats and *μ* was set to be 1.3 × 10^−8^ mutations per site per year (Ma and Bennetzen 2004).

### Gene Structure Annotation

We followed the MAKER-P pipeline (Campbell et al. 2014) to annotate gene models in the assembled genome using three classes of evidence: RNA-seq data, protein homology, and ab initio gene prediction (see supplementary text, Supplementary Material online). MAKER-P synthesized all information from these three different classes and produced final annotations with high evidence-based quality, requiring annotation evidence distance (which measures the goodness of fit of an annotation to the RNA/protein-alignment evidence supporting it) score <0.6. To assign gene functions, we followed the pipeline AHRD (https://github.com/groupschoof/AHRD) to automatically select the most concise, informative, and precise function annotation (see supplementary text, Supplementary Material online). For each gene, the associated gene ontology (GO) annotation(s) were assigned according to the predicted protein domain(s) (http://www.geneontology.org/external2go/interpro2go; last accessed January 9, 2019). To investigate the putative chromosomal locations of predicted genes in the genome, we performed whole-genome synteny alignment against the tomato genome using Satsuma v3.1.0 (Grabherr et al. 2010) and recorded the genes unambiguously associated with one identified syntenic region in tomato.

### Inference of Homologous and Orthologous Gene Clusters

We downloaded the annotated gene/protein-coding sequences of six other diploid Solanaceae species (including *S. lycopersicum*, *S. tuberosum*, *C.annuum*, *Nicotiana attenuata*, *Petunia axillaris*, and *P. inflata*) from the SolGenomics database (http://solgenomics.net) and used *Arabidopsis thaliana* as the outgroup species. The six Solanaceae species were chosen because they each have one well-assembled and annotated genome (i.e., contig/scaffold size >10 kb and gene completeness >95%) with a comparable number of annotated genes (∼35,000 genes). Identification of homologous gene clusters was performed using orthoMCL v2.0.9 (Li et al. 2003) with the default options. We obtained 3,103 single-copy 1-to-1 orthologous clusters that each contained one sequence from all seven species for downstream phylogenetic analyses. The coding sequences (CDS) of those 1-to-1 orthologs were aligned using PRANK v.150803 (Löytynoja and Goldman 2005) with codons enforced. As a quality check on all multiple sequence alignments, we removed poorly aligned regions using a sliding window approach that masked any 15-bp window from alignment if it had more than six mismatches (not counting indels/gaps, which were masked to N) among all the investigated species. After this process, any alignment with more than 20% of its sequence masked was removed from the analysis.

### Phylogenetic Analyses

We reconstructed evolutionary relationships between *Jaltomata* and the six other Solanaceous species for which we had whole-genome data. We used four different, but complementary, inference approaches to perform phylogenetic reconstruction: 1) maximum-likelihood applied to concatenated alignments (in RAxML v8.23; Stamatakis 2006), 2) consensus of gene trees (RAxML V8.23, with Majority Rule Extended [Salichos and Rokas 2013]), 3) quartet-based gene tree reconciliation (using ASTRAL v4.10.9) (Mirarab and Warnow 2015), and 4) Bayesian concordance of gene trees (using MrBayes v3.2 [Huelsenbeck and Ronquist 2001] followed by BUCKy v1.4.4 [Larget et al. 2010]) (see supplementary text, Supplementary Material online). Using these different tree reconstruction approaches allowed us to evaluate the extent to which they generated phylogenies that disagreed, as well as to identify the specific nodes and branches that were robust to all methods of phylogenetic reconstruction. In addition, we also reconstructed and annotated the chloroplast and mitochondrial genomes in *Jaltomata* and performed phylogenetic analyses of *J. sinuosa* with *S. lycopersicum*, *C. annuum*, and *N. attenuata* (the species for which chloroplast DNA and mitochondrial DNA sequences were readily available) using the concatenated sequences of mitochondrial or chloroplast genes in RAxML v8.23 (see supplementary text, Supplementary Material online).

Our phylogenetic analyses indicated that gene trees supported two conflicting bipartitions among *Jaltomata*, *Solanum*, and *Capsicum* (i.e., “[*Jaltomata*, *Solanum*], *Capsicum*” or “[*Jaltomata*, *Capsicum*], *Solanum*”) at roughly equal frequencies (see the Results section). To differentiate which of these topologies most likely represented the initial pattern of lineage splitting (i.e., the “true” species tree) rather than relatedness due to subsequent introgression among species, we compared the relative divergence times (node depths) among species, using sets of gene trees that each supported one of the two most conflicting bipartitions, with *N.attenuata* as the outgroup. Gene trees constructed from nonintrogressed (initial branching order) sequences are expected to have deeper mean divergence times at the two internal nodes (*T*_1_ and *T*_2_; fig. 3) than those constructed from introgressed sequences, because introgression will reduce sequence divergence between the two lineages that have exchanged genes (Fontaine et al. 2015). Internal node depths (divergence times) for each gene tree that supported one of these two alternative bipartitions were calculated from biallelic informative sites; per gene estimates were used to calculate the genome-wide means of divergence times *T*_1_ and *T*_2_, to determine which of the two topologies had higher average divergence times (see further, supplementary text, Supplementary Material online).

### Gene Family Analyses

To investigate changes in gene family sizes, we investigated the families that are significantly rapidly evolving among the six Solanaceae species including *Jaltomata*. (In this analysis, *C. annuum* was excluded because of the complex relationships among *Jaltomata*, *Capsicum*, and *Solanum*; see the Results section.) We determined the significantly expanded or contracted gene families along each branch of phylogeny using the program CAFE v3.0 (Han et al. 2013) with *P* value cutoff of 0.01. The input gene families were generated from the OrthoMCL program (Li et al. 2003). Phylogenetic relationships were based on the output from the RAxML analysis of the concatenated data set of 3,103 coding-sequence alignments. Divergence times among different species were directly retrieved from previous estimates among Solanaceae species (Särkinen et al. 2013). In addition, we further investigated the expansion of a specific candidate gene within *Jaltomata*, *SEUSS*, by examining gene trees, primary mapped Illumina reads (i.e., using only the best alignment of multimapped reads), PacBio reads spanning more than one gene copy, and expression patterns supported by RNA-seq from 14 *Jaltomata* species examined in our previous study (Wu et al. 2018) (see supplementary text, Supplementary Material online).

### Positive Selection Analyses

To infer positively selected genes in the *Jaltomata* genome, we tested 3,103 single-copy 1-to-1 orthologous genes (excluding *C. annuum*) whose gene tree topologies were the same as the inferred species tree. For each investigated gene, we inferred putative adaptive evolution (i.e., *d*_N_/*d*_S_ > 1) using the branch-site (BS) model (model = 2 and NS sites = 2) in PAML v4.4 (Yang 2007) on the terminal branch leading to *J. sinuosa.* This inference uses a likelihood ratio test to determine whether the alternative test model (fixed_omega = 0) is significantly better than the null model (fixed_omega = 1), and identifies putative positively selected genes as those with a likelihood ratio test *P* value <0.01 and a false discovery rate <0.2 (Benjamini and Hochberg 1995). Because multinucleotide mutations (MNMs) can cause false inferences of positive selection in the PAML BS test (Venkat et al. 2017), for all significant genes we further applied a more conservative BS model (BS + MNM) (Venkat et al. 2017) in which another parameter *δ* is incorporated to represent the relative instantaneous rate of double mutations to that of single mutations (Venkat et al. 2017). We then assessed how many and which BS-significant genes remained significant (*P *<* *0.01) in the BS + MNM test. A GO-enrichment analysis was performed on these remaining putative selected genes using ONTOLOGIZER v2.0 with the parent–child analysis and a cutoff *P* value of 0.01 (Bauer et al. 2008).

## Results

### Genome Assembly and Annotation

**Fig. 1.**
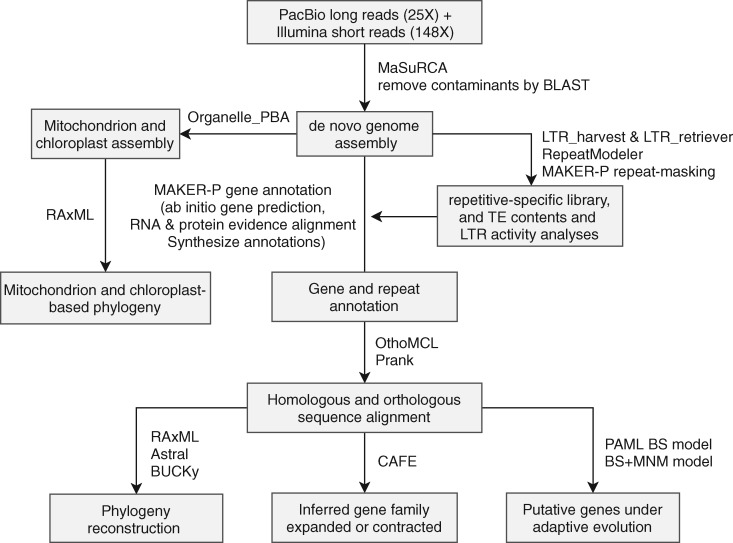
—Workflow of assembly of *J. sinuosa* genome and downstream comparative genomic analyses.

Following the workflow (fig. 1), we generated the first genome assembly of a *Jaltomata* species. The genome of *J. sinuosa* is estimated to be ∼1,512 Mb based on *k*-mer frequencies, which is consistent with the estimated size from flow cytometry (∼1,650 Mb; Haak DC, unpublished data). Using the MaSuRCA genome-assembly pipeline, we generated an assembly of ∼1,456 Mb, in which 96.6% of 1,515 BUSCO universal single-copy orthologous genes could be found (table 1). The assembly comprises 7,667 scaffolds, with a contig and scaffold N50 of 364.9 and 397.6 kb, respectively (table 1). In the ∼1,389 Mb (91.9%) of assembled reference genome that was also covered by at least five Illumina reads, only 68,203 sites had a base-call different from the base-call that was consistently indicated by the aligned Illumina reads (i.e., cases in which all Illumina reads support a site to be, for example, “A” rather than the base-call “T” in the assembly, indicating a potential error in the assembly; this criterion was applied to both mismatches and indels and excluded any sites where the Illumina reads indicated two different alleles at a site). This examination of base-call error rate across 91.9% of assembly, exclusively targeted for homozygous sites, suggested a lower bound of base-call error rate to be ∼0.005%. The upper bound of error rates was estimated to be 0.28% by counting the total discrepancies between all the primarily aligned reads and the assembled reference (including mismatches due to heterozygosity), although the error rate might be higher in genomic regions that are not well covered by Illumina data (Schmidt et al. 2017). Overall, the high coverage of the plant conserved single-copy genes, low base-calling error rate, and the high mapping rate of RNA-seq reads (96.2%, see below), indicate a high-quality assembly for the *J. sinuosa* genome. We also assembled the chloroplast genome into a single contig with a total length of 156 kb (Guanine–Cytosine [GC] content of 37.91%) with 82 annotated genes; the mitochondrial genome was assembled into a single contig with a total length of 317 kb (GC content of 42.05%) and 21 annotated genes.

**Table 1:** Summary of the *J. sinuosa* Genome Assembly

**Assembly features**	
Number of scaffolds	7,667
Genome size (Mb)	1,512
Assembly size (Mb)	1,454
Plant_CEGs (BUSCO) (%)^a^	87.0 (+8.7 + 0.9)
Contig N50 length (kb)	364.9
Scaffold N50 length (kb)	397.6
GC content %	37.94
RNA-seq mapped (%)^b^	90.6 (+5.6)
**Structure annotation**	
Numbers of protein-coding genes	34,726
Mean CDS length (bp)	1,101
Number of exons per gene	5.3

a Plant_CEGs (Clusters of Essential Genes) shows the percentage of complete single-copy orthologs plus the percentage of duplicated orthologs and fragmented orthologs.

b RNA-seq mapped indicates the percentage of uniquely mapped reads plus the percentage of multimapped reads.

### Repetitive Element Annotation and LTR Insertion Age Distribution

**Fig. 2.**
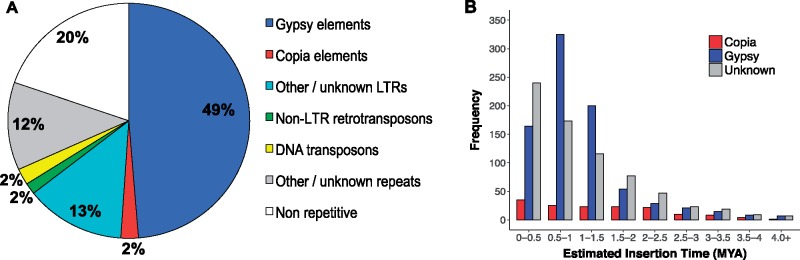
—Landscape of repetitive sequences in the *J. sinuosa* genome. (*A*) The repeat contents in the assembly. (*B*) The estimated insertion age distribution of full-length LTR-RTs in the assembly.

The assembled *J. sinuosa* genome contains a total of ∼1,158 Mb (80.29% of the assembly) of repetitive sequences (supplementary table S2, Supplementary Material online). LTR-RTs are the major source of repetitive sequences, accounting for 64.43% of the genome assembly (fig. 2*A*); of those LTR-RTs that could be unambiguously classified by LTR_retriever (∼190 Mb of genomic regions were annotated as unknown LTR-RTs; see supplementary text, Supplementary Material online), *Gypsy* elements are much more abundant (∼703 Mb) than *Copia* elements (∼34 Mb) (fig. 2*A* and supplementary table S2, Supplementary Material online). To evaluate the recent activity of LTR-RTs, we identified 1,682 full-length LTR-RTs within our data set (including 823 *Gypsy* and 151 *Copia*; supplementary table S3, Supplementary Material online) and, using the distribution of sequence divergence between the two terminal repeats within each LTR element, we infer a recent burst of *Gypsy* element activity around 1–2 Ma (fig. 2*B*). In comparison, the *C. annuum* and three *Solanum* genomes (*S. lycopersicum*, *S. tuberosum*, and *S. pennellii*) show many fewer recent insertions of *Gypsy* elements (supplementary fig. S1*B*–*D*, Supplementary Material online). Instead, *Gypsy* elements in *C. annuum* are inferred to have been most active around 3 Ma (supplementary fig. S1*A*, Supplementary Material online), whereas a higher abundance of recently active *Copia* elements was detected in *S. pennellii* (supplementary fig. S1*D*, Supplementary Material online), consistent with a previous study (Bolger et al. 2014).

### Gene Annotation and Transcription

A total of 34,726 high-confidence (annotation evidence distance <0.6) protein-coding genes with 37,106 transcripts were predicted (supplementary table S4, Supplementary Material online), which is similar to the 35,768 predicted genes in the domesticated tomato, *S. lycopersicum* (Tomato Genome Consortium 2012). The protein-coding sequences (CDS) in the annotated genes have an average length of 1,101 bp and the predicted genes have an average of 5.3 exons (supplementary table S5, Supplementary Material online); both are similar to the average CDS length (∼1,027 bps) and exon number (∼4.9) in the tomato annotation ITAG3.2 (Tomato Genome Consortium 2012). Through whole-genome synteny alignment, 23,013 (66.3%) of these predicted *Jaltomata* genes were associated with unambiguous syntenic regions within the tomato genome (supplementary table S6, Supplementary Material online). Among all annotated genes, nearly all of them (99.90%) were functionally annotated through the AHRD pipeline (supplementary table S7, Supplementary Material online). The transcriptome-wide RNA-seq reads (Wu et al. 2018) from *J. sinuosa* were mapped against the assembly with an alignment rate of 96.2%, with 67.81% of annotated genes having more than 0.5 transcripts per million. Because RNA-seq data from one species might only sample a subset of expressed genes, we also mapped the RNA-seq reads from 13 other *Jaltomata* lineages (Wu et al. 2018) back to the assembly and identified 82.83% of annotated genes that have transcripts per million >0.5 in at least one species. We identified 11,563 gene families that were shared among *J. sinuosa*, *S. lycopersicum*, *C. annuum*, *N.attenuate*, and *P. axillaris*, whereas a total of 953 gene families were specific to *J. sinuosa* (fig. 4*A*). GO term enrichment analysis indicated these *J. sinuosa*-specific genes are significantly over-represented in negative regulation of metabolic process or catalytic activity (supplementary table S9, Supplementary Material online).

### Complex Phylogenetic Relationships between *Jaltomata*, *Solanum*, and *Capsicum*

**Fig. 3.**
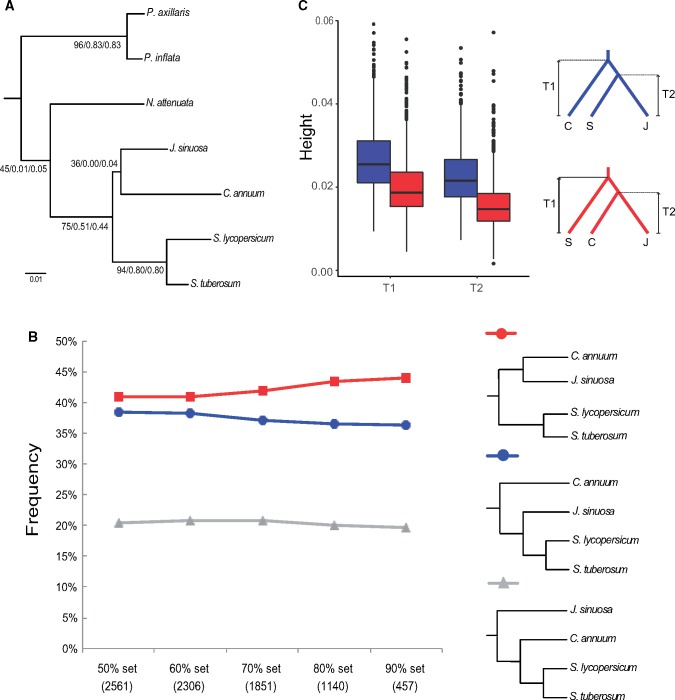
—Phylogenetic relationships among the seven investigated Solanaceae species. (*A*) Concatenated RAxML tree rooted by *A. thaliana*. The values above each internal branch indicate the proportion of gene trees supported (%), internode certainty, and tree certainty all supporting each node in the majority-rule consensus tree, from BUCKy (Larget et al. 2010). (*B*) The percentage of gene trees supporting the three alternative phylogenetic positions of *Jaltomata* relative to *S. lycopersicum*, *S. tuberosum*, and *C. annuum* (assuming *Solanum* is monophyletic). Five different sets of gene trees were used, which were selected based on the average bootstrap cutoff across the gene tree. (*C*) Evaluation of the relative node depths (ages) in the two alternative bipartitions among *Jaltomata* (J), *Solanum* (S), and *Capsicum* (C). The higher average height (depth) of *T*_1_ and *T*_2_ indicate that *Jaltomata* and *Solanum* are sister clades, whereas shallower gene trees supporting *Jaltomata* and *Capsicum* as sister are likely to be influenced by introgression.

The four phylogeny-reconstruction methods all supported the same topology among the seven Solanaceae species that included *J. sinuosa* as more closely related to *C. annuum* than to *S. lycopersicum* (fig. 3*A* and supplementary fig. S2, Supplementary Material online). However, there was also substantial gene tree discordance observed at this node: only 42% of gene trees supported the branch that groups *J. sinuosa* together with *C. annuum*. An internode certainty value of zero on this branch indicates that the number of gene trees supporting this bipartition is almost equal to the number of gene trees supporting the most common conflicting bipartition (fig. 3*A*). A similar pattern was also observed at the internode that splits the two *Petunia* species from the other investigated Solanaceae species (fig. 3*A*).

In order to further investigate the phylogenetic placement of *Jaltomata* relative to *Solanum* and *Capsicum*, we examined the proportion of gene trees supporting the each of the three different possible topologies, using five groups of genes which had progressively higher bootstrap support (i.e., average % bootstrap support across the RAxML gene trees of greater than 50%, 60%, 70%, 80%, or 90%). For the largest (>50% bootstrap) group, we found that ∼42% of gene trees supported the most common bipartition (i.e., [*Jaltomata*, *Capsicum*], *Solanum*), but that ∼38% gene trees supported the conflicting topology that places *Jaltomata* as sister to *Solanum*; this general pattern is consistent across sets of genes with increasingly higher power (fig. 3*B*). Our phylogenies constructed from the concatenated data of 10 mitochondrial genes supported *Jaltomata* as the sister clade of *Capsicum*, whereas the concatenated data from 72 chloroplast genes supported *Jaltomata* as closer to *Solanum* (supplementary fig. S3 and table S10, Supplementary Material online). However, there were very few (only four) informative sites in the mitochondrial data set that can unambiguously resolve the relationship (supplementary fig. S3*B*, Supplementary Material online), thus we interpret this apparent conflict between plastid genomes is due to a lack of power (too few informative variable sites) specifically in the mitochondria data.

To differentiate which of the two majority conflicting topologies is due to the initial lineage splitting events versus subsequent introgression, we compared the estimated divergence times among the three species from those genes supporting either of the two conflicting topologies. The gene trees supporting “*Solanum*, (*Capsicum*, *Jaltomata*)” have lower mean divergence times *T*_1_ and *T*_2_ relative to those from the gene trees supporting “*Capsicum*, (*Jaltomata*, *Solanum*)” (fig. 3*C*). Because introgression reduces sequence divergence between species exchanging genes (see the Materials and Methods section), our data suggest that the initial species branching order was “*Capsicum*, (*Jaltomata*, *Solanum*),” whereas the excess of gene trees supporting “*Solanum*, (*Capsicum*, *Jaltomata*)” is due to subsequent introgression between *Capsicum* and *Jaltomata*.

### Dynamic Evolution of Gene Families in *Jaltomata*

**Fig. 4.**
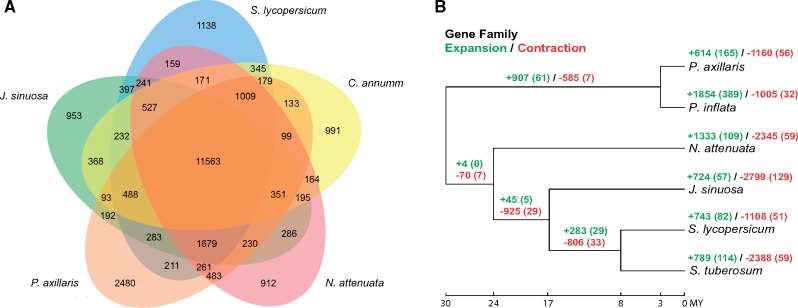
—Comparative gene family analyses. (*A*) Unique and homologous gene families. The numbers of unique or shared gene families are shown in the corresponding diagram components. (*B*) Gene family expansion and contraction patterns across the Solanaceae. The numbers of expanded (green) and contracted (red) gene families are shown in each branch, and numbers in the brackets are the number of significantly expanded or contracted gene families (*P *<* *0.01).

We identified 129 rapidly evolving gene families that contracted specifically on the branch leading to *J. sinuosa* (fig. 4*B*). Interestingly, of these we found a gene family (Cluster 7; supplementary table S11, Supplementary Material online) that is functionally involved in pollen–pistil interactions (GO: 0048544) for which there are only 21 genes in the *J. sinuosa* genome, whereas the other five investigated Solanaceae genomes have 38–46 genes (supplementary table S12, Supplementary Material online). This contracted gene cluster included receptorlike kinase family proteins with an S-locus glycoprotein domain, which are involved in plant reproduction and signaling in pollen–pistil interactions (supplementary table S11, Supplementary Material online). Based on our analysis of syntenic blocks with the tomato genome, the genes in this cluster are distributed across multiple chromosomes in the genome (supplementary table S11, Supplementary Material online). The loss of multiple putative S-locus receptor kinase family proteins is consistent with the ancestral loss of self-incompatibility in all *Jaltomata* lineages (see the Discussion section). Compared with *Jaltomata*, contraction of different gene families was detected in the *S. lycopersicum* and *N. attenuata* genomes (supplementary table S13, Supplementary Material online).

**Fig. 5.**
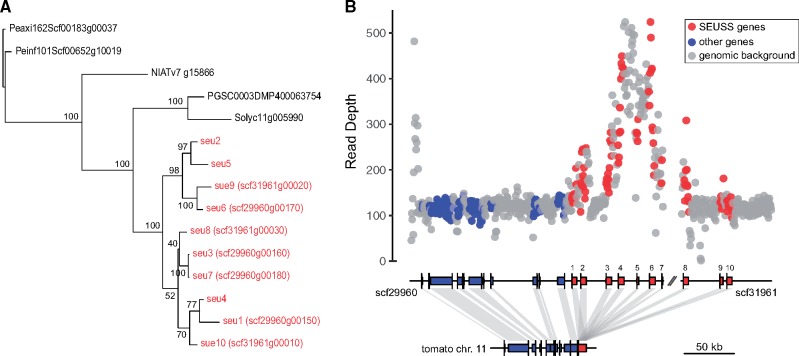
—Evolutionary dynamics of copy number of the gene *SEUSS* in *J. sinuosa*. (*A*) Lineage-specific expansion of *SEUSS* in *J. sinuosa*. The ten duplicated copies were labeled according to their relative position order along the located scaffolds. Among them, seven copies were annotated by the MAKER pipeline. (*B*) Validation that there are multiple copies of *SEUSS* using read depth of Illumina DNA-seq reads which is at least equal or above the genomic background and adjacent single-copy genes. Each dot represents read depth from a 1 kb sliding window. The red dots indicate the windows that overlapped with *SEUSS* genes, whereas the blue dots indicate the windows that overlapped with other single-copy genes. The gray dots are the windows that contain other (intergenic) regions.

We also identified 57 rapidly evolving gene families that expanded specifically on the branch leading to *J. sinuosa* (fig. 4*B*). These gene families were involved in a broad range of functions, including genes responsible for plant stress-related response, such as disease resistance, response to wounding, and regulation of nitrogen compound metabolic process (supplementary table S14, Supplementary Material online). Similar to *J. sinuosa*, significant lineage-specific expansion of various stress-related gene families was also found in other Solanaceae species, including genes involved in heat shock, oxidative stress, and resistance to fungi in *S. lycopersicum* and *N. attenuata* genomes (supplementary table S15, Supplementary Material online). Of the expanded families specific to the *Jaltomata* genome, one particularly interesting example involved the transcription factor *SEUSS*, which is known to play an important role in floral organ development in model systems (see the Discussion section). Although only one copy of *SEUSS* was identified in each other Solanaceae genome, an estimated ten copies (seven of them were annotated by the MAKER pipeline) were detected in the *J. sinuosa* genome (fig. 5*A*), and the gene tree of these copies indicates the expansion of *SEUSS* happened recently and specifically in the *Jaltomata* lineage (fig. 5*A*). The inferred ten copies of *Jaltomata SEUSS* gene were found on two scaffolds, suggesting the expansion of *SEUSS* gene copy number is due to recent tandem duplication (fig. 5*B*).

Several methods validated the presence of >1 *SEUSS* copies in *Jaltomata*. We identified five PacBio long reads spanning two adjacent *SEUSS* copies. We also found that read depth around each *SEUSS* copy was equal to or higher than read depth in the adjacent single-copy loci or background genomic regions (fig. 5*B*). The higher depth of primary mapped reads at some of these inferred *SEUSS* copies suggests that there might be additional paralogous copies of these loci that we were unable to differentiate because their sequences are too similar (i.e., due to very recent duplication events). Using RNA-seq data from Wu et al. (2018), we found that four structurally intact copies (*SEUSS* 4, 6, 8, and 10) have >25 reads that uniquely map to them in at least one of 13 *Jaltomata* species, suggesting there are at least four putatively functional copies in the genus (supplementary table S16, Supplementary Material online). Across all 13 species, only RNA-seq reads from the sampled reproductive, but not vegetative, tissues mapped to *SEUSS* loci (supplementary table S16, Supplementary Material online), and expression of *SUESS* copies varied among species: three copies (*SEUSS* 4, 6, and 8) were expressed in all purple-fruited lineages (i.e., *J. repandidentata*, *J. procumbens*, and *J*. *darcyana*), whereas almost all of the ten inferred *SEUSS* copies had no reads mapped (i.e., very low expression) in most of the orange/green-fruited lineages (including *J. sinuosa*) (supplementary table S16, Supplementary Material online). This difference in number of reads mapped in different *Jaltomata* lineages cannot be explained by sequence divergence, because the purple-fruited lineages are more distant from *J. sinuosa* relative to other investigated *Jaltomata* lineages.

### Detection of Genes Potentially under Positive Selection in *Jaltomata*

Using the BS model in PAML, we identified 89 genes out of 6,582 testable genes as putatively under positive selection (i.e., the lineage-specific selection model fit significantly better than the null model, *P* value <0.01 and false discovery rate <0.2; supplementary table S17, Supplementary Material online). After implementing the BS + MNM test (Venkat et al. 2017) on these 89 putative selected genes, 58 of them remained significant (*P* value <0.01; supplementary table S16, Supplementary Material online). Some of these 58 loci are involved in stress responses, including resistance to osmotic and oxidative stress, response to heat shock, and heavy metal transportation (supplementary table S17, Supplementary Material online); however, these putatively selected genes were not enriched for any specific GO terms (supplementary table S18, Supplementary Material online). Nonetheless, among these loci are several candidates for elements of *Jaltomata*-specific trait evolution, including loss of self-incompatibility, growth during (floral) development, and regulation of pigment biosynthesis (see the Discussion section).

## Discussion

Here, we generated a high-quality genome sequence of a representative species (*J. sinuosa*) from within *Jaltomata*, a rapidly evolving, florally and reproductively diverse genus in the Solanaceae. We used these data to clarify a complex history of origin that *Jaltomata* shares with its two most closely related genera—*Solanum* and *Capsicum*—and to infer recent TE dynamics that might be responsible for genome-size evolution within the Solanaceae. We also identified overall patterns of significant gene family gain and loss, as well as adaptive molecular evolution, which could be implicated in the rapid reproductive trait evolution that is distinctive to *Jaltomata* among its Solanaceous relatives.

### Comparative Phylogenomic Analysis Reveals Complex History of Divergence among *Jaltomata* and Its Closest Relatives

The Solanaceae is a highly speciose plant family, with an estimated 100 genera and 2,500 species that have all evolved within the last ∼30 Myr. Previous phylogenetic studies have confirmed that many lineages within the Solanaceae arose within a highly compressed time frame (Olmstead et al. 1999; Särkinen et al. 2013), making resolution of some evolutionary relationships challenging. In particular, previous molecular phylogenetic studies using chloroplast and nuclear loci indicated that *Jaltomata* is close to both *Solanum* and *Capsicum*, however the relationship among these three genera has varied depending upon the specific loci used in phylogenetic reconstruction (Bohs and Olmstead 1997; Olmstead et al. 1999; Walsh and Hoot 2001; Olmstead et al. 2008; Särkinen et al. 2013). The *J.sinuosa* genome therefore provided an opportunity to evaluate and clarify the historical evolutionary relationships among key genera within Solanaceae.

Just as with other recent phylogenomic studies of contemporary (Brawand et al. 2014; Lamichhaney et al. 2015; Novikova et al. 2016; Pease et al. 2016) or more ancient rapid radiations (Jarvis et al. 2014; Wickett et al. 2014; Suh et al. 2015; Yang et al. 2015), we detected evidence for substantial gene tree discordance in relationships among the seven Solanaceous species analyzed here. This included high discordance (internode uncertainty) at the internode that split the *Petunia* lineages from the remaining species (fig. 3), as well as the internodes separating *Jaltomata*, *Solanum*, and *Capsicum*. In particular, our concatenation-maximum-likelihood phylogeny supported a closer relationship between *Jaltomata* and *Capsicum*, but we also detected a similar number of individual gene trees supporting the alternative topology of *Jaltomata* and *Solanum* as more closely related (fig. 3). Genome-wide, the observed pattern of minority gene tree discordance was not consistent with the action of incomplete lineage sorting (ILS) alone (under ILS the two alternative minority trees are expected to be approximately equally represented) (Degnan and Rosenberg 2009), indicating that a substantial component of discordance was likely also due to introgression between lineages (Huson et al. 2005).

Following the logical framework in Fontaine et al. 2015, in which the younger (shallower) tree topology is inferred to be due to introgression, we used the relative depth (age) of the two alternative tree topologies to infer that *Jaltomata* is likely sister to *Solanum* (a relationship supported by the tree with the older/deeper mean node depths). In contrast, an excess of gene trees supporting a sister relationship between *Jaltomata* and *Capsicum* (the tree with on average shorter/younger node depths) is likely to be due to introgression since the split of the three species; this is despite the observation that the latter tree is marginally more frequent across all gene trees compared with the next most common bipartition (fig. 3). Our use of whole-genome sequence data from *Jaltomata* therefore enabled us to disentangle the likely complex history of origin at the base of the clade that unites these three lineages.

Our analyses also highlighted the extensive phylogenetic incongruence among these and other Solanaceae genera more generally, a history that should be accounted for in comparative studies. In particular, assessing the level and distribution of incongruence is critical when making inferences about trait evolution in radiating lineages, as substantial gene tree discordance can contribute to incorrect inferences of convergence (“hemiplasy”) at both the phenotypic and molecular level (Hahn and Nakhleh 2016; Wu et al. 2018).

### TEs Contribute to Genome-Size Evolution across the Solanaceae

We also used the *J. sinuosa* genome assembly to examine possible causes of genome-size evolution, specifically variation in TE history. The estimated genome size of *J. sinuosa* (∼1.5 Gb) is >50% larger than the tomato genome (∼0.9 Gb), but less than half of the hot pepper genome (∼3.5 Gb), despite no accompanying change in ploidy level, suggesting that TEs might be an important contributor to genome-size variation in the Solanaceae. Indeed, we determined that nearly 80% of the assembly consists of repetitive elements, with the vast majority belonging to the *Gypsy* superfamily of LTR-RTs (fig. 2). We also inferred a recent proliferation of *Gypsy* elements that occurred around 1–2 Ma, which might have contributed to the larger genome size in *Jaltomata* lineages relative to *Solanum*. Previous studies have revealed a similar pattern of relatively recent *Gypsy* proliferation in other Solanaceae species, including a substantial excess of *Gypsy* elements (12-fold more frequent than *Copia* elements) within the hot pepper genome compared with domestic tomato (Kim et al. 2014). Similarly, genome-size variation scales with variation in the number of *Gypsy* repeats among four *Nicotiana* species (Xu et al. 2017). In contrast, within *Solanum*, the larger genome size of wild species *S. pennellii* compared with domesticated tomato is associated with a recent proliferation of *Copia*-like elements (Bolger et al. 2014). Overall, this and other recent analyses indicate that differential activity of LTR-RTs contributed substantially to differences in genome size across the Solanaceae and suggest that future studies examining these and broader TE activity and DNA loss (Kapusta et al. 2017) could reveal important dynamics of genome-size and content variation in this group.

### Both Gene Family Evolution and Specific Molecular Changes Contribute to Unique Reproductive Trait Variation within *Jaltomata*

Unlike close relatives *Solanum* and *Capsicum*, species in *Jaltomata* exhibit extensive floral diversity in corolla shape and nectar volume and color (Miller et al. 2011) in addition to an apparently ancestral transition to self-compatibility (Mione 1992) (Kostyun and Mione, unpublished data). Another goal in developing a representative genome sequence for *Jaltomata* was therefore to identify genetic mechanisms that might have contributed to the evolutionary origin of these derived floral and reproductive states. In our previous phylogenomic study using transcriptome data, we investigated patterns of molecular evolution in several thousand loci across 14 species within *Jaltomata* and found that relatively few of the genes that showed a signal of positive selection (i.e., with *d*_N_/*d*_S_ > 1) had functional associations with floral development. However, extensive gene tree discordance and very low sequence divergence (<1% among the species in the radiating group that displays the derived floral traits) only allowed a small subset of genes to be tested at some internal branches (Wu et al. 2018). Moreover, this transcriptome-based study only focused on molecular variation in coding sequences, although regulatory changes and structural variation, such as gene duplications, are also known contributors to the evolution of novel traits (Hoekstra and Coyne 2007). The generation of a reference genome in this group therefore allowed us to address the possible role of additional genetic variation that could not be investigated previously.

Interestingly, a major inference from our whole-genome analysis is that each of the seven Solanaceae lineages examined has experienced substantial expansion and contraction of gene families (fig. 4*B*), and several of these might have contributed to the distinctive reproductive trait evolution in *Jaltomata*. First, we detected significant contractions in gene families functionally associated with pollen–pistil interactions, including the putative S-locus receptor kinase family proteins, which could be a genomic signature of an ancestral transition to self-compatibility in this genus. Within the Solanaceae, self-incompatibility is mediated by the “S-locus” which encodes a single female/stylar S-determinant (i.e., S-RNase) and one or more pollen-expressed F-box proteins (Iwano and Takayama 2012) that interact to cause pollen rejection when the male and female parent share the same allele at the S-locus (McClure et al. 1989). Multiple studies have shown that loss of the pistil-side S-RNase mediates the transition from SI to SC in Solanaceous species, often followed by the subsequent loss of one or more pollen-side F-box proteins and other components of the SI-machinery (Charlesworth and Charlesworth 1979; Stone 2002; Takayama and Isogai 2005; Covey et al. 2010). Similarly in Brassicaceae, breakdown of SI appears to occur via loss of pistil-side factors, although the specific genetic mechanisms controlling SI are distinct between these two families (Kusaba et al. 2001; Sherman-Broyles et al. 2007; Tang et al. 2007; Shimizu et al. 2008). Several analyses in both these families have documented genome-wide effects of loss of SI (Hu et al. 2011; Slotte et al. 2013), however in most cases the associated transition to selfing has been recent. In contrast to these studies, all examined *Jaltomata* species are self-compatible (Mione 1992) (Kostyun and Mione, unpublished data), indicating that the loss of self-incompatibility occurred early within *Jaltomata*, prior to the origin of the major subgroups within this clade (i.e., >3 Ma). Consistent with an early loss of SI in this genus, we found that heterozygosity in each of 14 *Jaltomata* species (inferred by remapping RNA-seq reads from Wu et al. [2018] to the genome assembly) is comparable to that found in self-compatible species of *Solanum*, and much lower than in self-incompatible species of *Solanum* (Pease et al. 2016) (see supplementary table S8, Supplementary Material online). This early loss of SI might explain why we identified significant contractions of gene families related to pollen–pistil interactions specifically in *Jaltomata*. A similar pattern was observed in the genome of *Caenorhabditis nigoni*—a self-fertile nematode species that split from its outcrossing sibling species *C. briggsae* ∼3.5 Ma (Thomas et al. 2015)—in which several hundred genes mediating protein–protein interactions, including the genes important for sperm–egg interactions, appear to have been lost (Yin et al. 2018).

Unlike the contraction of gene families potentially associated with relaxed selection, we also identified expansion of gene families that might be associated with the evolution of novel floral traits. In particular, we detected recent tandem duplications of one interesting candidate gene *SEUSS* (fig. 5). *SEUSS* is a transcription factor, which in *A. thaliana* interacts with *LEUNIG* within a transcriptional corepressor complex to negatively regulate the expression of homeotic MADS-box gene *AGAMOUS* (Franks et al. 2002, 2006; Sridhar et al. 2006). Mutations in *SEUSS* cause ectopic and precocious expression of *AGAMOUS* mRNA, leading to partial homeotic transformation of floral organs in the outer two whorls (i.e., sepal and petal) (Franks et al. 2002). *LEUNIG* and *SEUSS* also have a more general role in lateral organ patterning and boundary formation in *Arabidopsis*, possibly through interactions with transcriptional factors in the *YABBY* and *KNOX* gene families (Stahle et al. 2009; Lee et al. 2014).

These functional roles are especially interesting with respect to the origin of floral diversity in *Jaltomata*, as the two derived corolla shapes (i.e., campanulate and tubular) are formed due to differential accelerated growth rates and variation in petal fusion (i.e., boundary formation) (Kostyun et al. 2017). Thus, the function of *SEUSS* is closely related to the mechanism generating the corolla shape diversity in *Jaltomata*. In general, duplication and divergence of floral identity genes appear to play an important role in the evolution of floral morphology in plants, such as the expansion of MIKC^c^-type MADS-box genes (Hileman et al. 2006; Panchy et al. 2016). Although we do not yet have expression data for individual tissues (such as individual floral organs), we found that *SEUSS* copies were expressed only in the reproductive tissues of some lineages, and that species differ in the expression of these *SEUSS* copies. Interestingly, the *SEUSS* genes showed more limited expression in the orange-fruited lineages (supplementary table S15, Supplementary Material online) with the derived floral traits (i.e., higher extent of corolla fusion), suggesting that downregulation of some or all copies of this negative regulator of floral development might have contributed to the evolution of novel floral (corolla) morphs in this group. Future work with tissue-specific expression and function across different *Jaltomata* species with distinct corolla shapes will allow us to further assess whether *SEUSS* variants are strongly implicated in floral trait divergence across *Jaltomata*.

Apart from gene family contractions and expansions, we also detected loci with patterns of adaptive molecular evolution that could be functionally implicated in reproductive trait changes. Although many of the 58 genes that are inferred to be under positive selection specifically within the *Jaltomata* lineage (supplementary table S16, Supplementary Material online) are involved in general plant processes, some are from functional classes associated with the unique reproductive trait variation observed within this clade. For example, several candidates are associated with pollen–pistil interactions, including an S-ribonuclease binding protein that mediates the degradation of self-pollen (Sims and Ordanic 2001; Kao and Tsukamoto 2004). Novel changes in these proteins might have followed the early loss of self-incompatibility in this genus, perhaps as a result of reduced constraint on previous functions and the adoption of new roles under the altered reproductive environment of self-compatibility. Other potentially interesting candidates include an auxin response factor, a *WRKY* transcription factor, and a *NAC* transcription factor, all of which function in multiple plant developmental processes (Johnson and Lenhard 2011). Although the plant *NAC* gene family is quite large and its members play key roles in numerous developmental and stress response pathways, this putative candidate is especially interesting as *NAM* (a *NAC* protein) has been shown to function in organ boundary specification, including in flowers of closely related *Petunia* (Souer et al. 1996; Zhong et al. 2016), and development of the novel floral forms in *Jaltomata* appears to be specifically associated with altered floral organ boundaries (Kostyun et al. 2017).

### The *Jaltomata* Genome as a Tool for Future Comparative Analysis

Overall, the generation of a high-quality *Jaltomata* genome enabled us to evaluate genetic changes specific to this diverse lineage and across the Solanaceae more generally, as well as to clarify the historical evolutionary relationships among these important clades. In doing so, we inferred that *Jaltomata* has a complex history of divergence from its most closely related genera, which involves substantial introgression following initial lineage divergence. Based on quantification of recent TE activity, we infer that TE dynamics are likely an important contributor to genome content and genome-size differences among groups in the Solanaceae. Using comparisons of gene family expansion and contraction, and more conventional analyses of adaptive protein evolution, we identified several genetic changes that might have either facilitated or accompanied the unique reproductive trait evolution observed in this clade.

In the future, the interesting evolutionary features and central phylogenetic placement of *Jaltomata* within the Solanaceae offer a unique opportunity to examine patterns of coding, structural, and TE evolution across this diverse and speciose plant family, as well as to further evaluate mechanisms that could contribute to the rapid reproductive diversification observed specifically within *Jaltomata*. These future comparative genomic analysis could reveal additional large- and small-scale genetic changes responsible for genomic differentiation and divergent form and function among a set of economically important clades that are well-established models for developmental biology (*Petunia*), reproductive interactions (*Nicotiana*), secondary metabolite production (e.g., *Capsicum*), and ecophysiological responses (e.g., *Solanum*), as well as within this emerging model for analyzing the evolution of novel floral evolution.

## Supplementary Material

Supplementary data are available at *Genome Biology and Evolution* online.

## Supplementary Material

Supplementary Data

## Notes

**Data deposition:** The project has been deposited at NCBI BioProject database under the accession number PRJNA474403
