# Supplementary material for: Genome Sequence of Jaltomata Addresses Rapid Reproductive Trait Evolution and Enhances Comparative Genomics in the Hyper-Diverse Solanaceae
Source: Genome Biol Evol. 2019 Jan 4;11(2):335–49. doi: 10.1093/gbe/evy274 (PMC6368146; doi:10.1093/gbe/evy274)
Supplement: Supplementary Data [file evy274_supp.zip › Supplementary information_rv3_Oct4.docx]

Supplementary Information for: Genome sequence of *Jaltomata* addresses rapid reproductive trait evolution and enhances comparative genomics in the hyper-diverse Solanaceae

*Meng Wu, Jamie L. Kostyun, and Leonie C. Moyle*

**Contents**

Estimation of Base-Call Error Rate in the Assembly 2

Estimating Heterozygosity in *Jaltomata* Species 2

Classification and Inferring the Timing of Recent LTR-RT Activity 2

Gene Structure Annotation 3

Gene Functional Annotation 3

Phylogenomic Analyses 4

Phylogenetic Analysis of Genes in Organelle Genomes 4

Differentiating Introgression Phylogeny from Species Tree 4

Examination of SEUSS Gene Copies Within *Jaltomata* 5

Supplementary Figures 6

Literature Cited 9

**Estimation of base-call error rate in the assembly**

To obtain an upper bound on the error rate, we calculated the total base discrepancies (including mismatches due to heterozygosity) between the primarily aligned individual Illumina reads and the long-read assembly using Qualimap2 (Okonechnikov et al. 2016). To obtain a lower bound of error rate estimate, we mapped Illumina short reads back to the assembly by BWA v.0.7.12 (Li and Durbin 2010) and called variants with SAMtools v.0.1.19 (Li et al. 2009). Assembly error rates were estimated by dividing the number of detected base-call variants (i.e. sites with a nucleotide consistently supported by all Illumina reads but different from that in the assembled reference) and indels by the total length of covered genomic regions, at sites with mapping quality of more than 20 and mapped read depth ≥ 5. A potential error is indicated when all short reads support a site to be, for example, ‘A’ whereas the base-call is ‘T’ in the assembly. Conversely if all short reads agree with the site in the assembly, a correct base-call was inferred. Because a site was included only if all short reads supported the same base call, this lower bound error estimate was targeted to homozygous sites; any site where the short read data was ambiguous (two alternative alleles were detected, e.g. T and A) was excluded from the analysis. Moreover, because the Illumina read depth was on average ~100 reads, the large majority of the sites included in this lower bound estimate had substantially in excess of 5 reads; the probability that a genuinely heterozygous site appears to be homozygous based on these short reads is very small when the number of short reads increases above 5.

**Estimating heterozygosity in *Jaltomata* species**

To estimate the average heterozygosity in *J. sinuosa* and 13 other *Jaltomata* species that were previously investigated with transcriptome data (Wu et al. 2018), we mapped these RNA-seq reads from each of the 14 species to the *Jaltomata* genome assembly using STAR v2.5.2 (Dobin et al. 2013). SAM files generated were converted to sorted BAM files using SAMtools v. 0.1.19 (Li et al. 2009). SAMtools *mpileup* was then used to call alleles from the BAM files for all lineages, requiring non-reference allele calls to have Phred sore ≥ 30 and mapped read coverage ≥ 10. Heterozygosity was estimated by dividing the number of heterozygous sites by the total number of sites. Note that the estimates of heterozygosity here and in the previous analysis of RNAseq data are based on sequences from a single (heterozygous) reference individuals in each accession, so may be an underestimate of species-wide heterozygosity. Consistent with an early loss of SI in this genus, we found that the heterozygosity of all the 14 investigated *Jaltomata* species (Table S8) is comparable to that estimated from single heterozygous reference individuals from self-compatible species of *Solanum* but much reduced relative to that estimated from single heterozygous reference individuals from self-incompatible species of *Solanum* (Pease et al. 2016).

**Classification and inferring the timing of recent LTR-RT activity**

The predicted full-length LTR-RTs from LTR-harvest were classified through a module in LTR-retriever (Ou and Jiang 2018), which categorizes LTR-RTs as Gypsy or Copia based on the specific order of protein domains. The ‘unknown’ category represents the group of elements that is ambiguous based on the predicted protein domains for each of these elements; that is, these are cases in which the order of domains is ambiguous, either because the LTR-RT 1) lacks of one or more protein domains (though they retain the two terminal repeats); or 2) has ambiguous protein domains (e.g. some Copia-like LTR-RTs appear to contain a Gypsy-like conservative domain, potentially due to nested insertion events) that are therefore unable to be classified. Our estimates of the timing of specific bursts of LTR-RT activity were based on the distribution of sequence divergence between terminal repeats of each full-length LTR-RT, as estimated in LTR-retriever (Ou and Jiang 2018). Briefly, insertion time (T) of each full-length LTR-RT was estimated as T = K/2μ, where K is the divergence rate between the two terminal repeats and μ was set to be 1.3 × 10^-8^ mutations per site per year (Ma and Bennetzen 2004).

**Gene structure annotation**

We used three different classes of evidence--RNA-seq data, protein homology, and *ab initio* gene prediction--in the MAKER2 pipeline v2.31.9 (Campbell et al. 2014) to annotate gene models. Assembled transcripts were generated from RNA-seq data sampled from multiple vegetative and reproductive tissues of the same accession of *J. sinuosa*, which are described in our previous phylogenomic study (Wu et al. 2018). Homologous protein sequences were downloaded from the SwissProt *Solanaceae* protein dataset (Magrane and Consortium 2011). For *ab initio* gene predictions, we used three programs SNAP (Korf 2004), GeneMark-ES (Lukashin and Borodovsky 1998) and AUGUSTUS v3.2.3 (Stanke et al. 2006). Each gene model was predicted using a two-pass (iterative) MAKER2 workflow. The initial SNAP HMMs were generated by CEGMA (Parra et al. 2007). After that, we ran the MAKER-P first round with the generated SNAP and GeneMark HMMs along with other evidence sets (i.e. transcriptome data and aligned *Solanaceae* protein set), while setting the parameters as “est2genome=1” and “protein2genome=1”. The gene predictors were then retrained one additional time, with the parameter setting as “est2genome=0” and “protein2genome=0”. The results from the MAKER-P *ab-initio* gene predictions were converted to the updated SNAP and AUGUSTUS HMMs, which were used for the second round of MAKER-P.

**Gene functional annotation**

To assign gene functions, we first conducted sequence homology searches using BLASTP on the predicted protein sequences, against different protein datasets from SwissProt protein knowledgebase and its supplement TrEMBL (Magrane and Consortium 2011), Arabidopsis genome annotation TAIR10 (Lamesch et al. 2012), and tomato genome annotation ITAG3.2 (The Tomato Genome Consortium 2012). Putative protein domains on protein sequences were identified using InterProscan v5.25 (Jones et al. 2014). Finally, we used the pipeline AHRD (<https://github.com/groupschoof/AHRD>) to automatically select the most concise, informative and precise function annotation, with SwissProt, TAIR10, ITAG2.4, and TrEMBL being scored with different database weights (100, 50, 50, 10, respectively).

**Phylogenomic analyses**

Four different approaches were used to perform phylogenetic reconstruction: 1) maximum-likelihood (ML) applied to concatenated alignments; 2) consensus of gene trees; 3) quartet-based gene tree reconciliation; and 4) Bayesian concordance of gene trees. The ML concatenation tree was inferred by using the GTRGAMMA model in RAxML v8.23 with 100 bootstraps (Stamatakis 2006). The consensus tree with internode certainty (IC) and tree certainty all (TCA) support scores were also generated using RAxML with the option for Majority Rule Extended (Salichos and Rokas 2013). The quartet-based estimation of the species tree was inferred by using the program ASTRAL v.4.10.9 with 100 bootstraps (Mirarab and Warnow 2015). Finally, the Bayesian primary concordance tree and associated concordance factors (CFs: IC and TCA) at each internode of the primary concordance tree was computed in the program BUCKy v1.4.4 (Larget et al. 2010). The input of a posterior distribution of gene trees was generated from an analysis with MrBayes v3.2 (Huelsenbeck and Ronquist 2001). We ran MrBayes for one million Markov chain Monte Carlo (MCMC) generations, and every 1000th tree was sampled. After discarding the first half of the 1000 resulting trees from MrBayes as burnin, BUCKy was performed for one million generations with the default prior probability (*α* = 1) (Larget et al. 2010). All inferred species trees were plotted using the R package “phytools” (Revell 2012).

**Phylogenetic analysis of genes in organelle genomes**

We used the pipeline “Organelle_PBA” to reconstruct the chloroplast and mitochondrion genomes in *Jaltomata* using PacBio reads (Soorni et al. 2017). During the assembly, the chloroplast genome of *Solanum lycopersicum* (GI: 007898.3) and the mitochondria genome of *Nicotiana tabacum* (GI: 56806513) were used as the reference genome to filter PacBio sequencing data. Genes on the chloroplast and mitochondrion genomes were then annotated using DOGMA (Wyman et al. 2004) and Motify (Alverson et al. 2010), respectively. We downloaded the chloroplast and mitochondrial protein-coding gene sequences of *S. lycopersicum*, *C. annuum*, and *N. attenuata* from NCBI (Table S19). The orthologous genes were aligned using PRANK v.150803 (Löytynoja and Goldman 2005). The phylogenetic tree of chloroplast and mitochondrion were reconstructed with the concatenated sequences using maximum likelihood with the GTRGAMMA model in RAxML v8.23 (Stamatakis 2006).

**Differentiating introgression phylogeny from species tree**

To differentiate which topology among *Jaltomata*, *Capsicum*, and *Solanum* most likely represented the initial pattern of lineage splitting (i.e. the ‘true’ species tree) versus subsequent introgression among species, we compared the relative divergence times (node depths) among species using gene trees that supported these two most conflicting bipartitions, with *Nicotiana attenuata* as the outgroup. In a rooted three-taxon phylogeny (i.e. “((P1, P2), P3), O” where P1, P2, and P3 are either *J. sinuosa*, *S. lycopersicum* or *C. annuum* and O is the outgroup *N. attenuata*), there are two divergence times: the earlier time (T1) when the first taxon P3 diverges from the remaining sister pair and the time T2 when the paired taxa P1 and P2 diverge (Fig. 3C, main text). We estimated T1 and T2 from biallelic informative sites, where allelic patterns can be represented as combinations of ancestral alleles (A) and derived alleles (B). T2 was calculated as: $T2=\frac{1}{N} (\frac{nABAA+nBAAA}{2})$ and T1 was calculated as $T1=\frac{1}{N} (\frac{nABAA+nBAAA}{2}+nBBAA)$, in which *N* is the number of sites (Fontaine et al. 2015). When P3 is the source of introgression, T2 is predicted to be lower which represents the time of introgression. When P1 or P2 is the source of introgression, both T1 and T2 will be lower. It is because that the true T2 will instead be the observed T1 and the time of ingression will be the observed T2.

**Examination of SEUSS gene copies within *Jaltomata***

Because our gene family analysis detected one important transcription factor locus *SEUSS* that showed rapid expansion specifically on the branch leading to *Jaltomata*, we further examined this apparent recent evolution of multiple copies of *SEUSS* genes in the *Jaltomata* genome, and their expression in different *Jaltomata* lineages. Using the tomato *SEUSS* gene in a homologous search, ten partial or complete copies of *SEUSS* (including seven copies annotated by the MAKER-P pipeline) were identified on two scaffolds (*scf29960* and *scf31961*) of *J. sinuosa* genome. Only a single copy was identified in each of the six other *Solanaceae* species. To confirm that the putative duplication events occurred after the split of Jaltomata from the other species/genera analyzed, we generated the gene tree of *SEUSS* among those *Solanaceae* species using maximum likelihood with the GTRGAMMA model in RAxML v8.23 (Stamatakis 2006), and showed that all inferred duplicate copies from Jaltomata were grouped within this tree (Fig. 5A, main text). We took several approaches to confirm the existence of multiple copies of *SEUSS* (i.e., to exclude assembly error). First, we searched for PacBio reads that spanned any two copies of identified *SEUSS* loci. Second, we examined the read depth of the Illumina short reads across the two relevant scaffolds to evaluate whether the read depth around each *SEUSS* copy was equal to or higher than read depth of the adjacent single-copy loci and genomic background. In cases where read depth is higher, this is likely due to an excess of multi-mapped reads at loci that have experienced very recent additional duplications that therefore cannot be distinguished during assembly. Third, using existing RNA-seq data from vegetative (seven tissues) or reproductive (four tissues) tissue pools among 13 different *Jaltomata* lineages (Wu et al. 2018), we examined whether each copy of the *SEUSS* locus was expressed or not in each tissue pool in each species. The program featureCounts (Liao et al. 2013) was used to assign either uniquely or multi-mapped reads to the genic regions of *SEUSS* from the generated input BAM files. Uniquely mapped reads were used to distinguish the expression of specific *SEUSS* gene copies in each species, especially at structurally-intact *SEUSS* copies.

**Figure S1.** The estimated LTR-element insertion age distributions in **(A)** *C. annuum* (pepper), **(B)** *S. tuberosum* (potato), **(C)** *S. lycopersicum* (tomato), and **(D)** *S. pennellii*.

**Figure S2.** Whole-transcriptome phylogeny inferred by four different approaches on 3,103 single-copy 1-to-1 orthologs. **(A)** Whole-dataset concatenated phylogeny (RAxML). **(B)** Majority rule phylogeny (RAxML) with IC/TCA scores. **(C)** Best-likelihood quartet-based phylogeny (ASTRAL) inferred from 100 replicates. **(D)** Primary concordance phylogeny produced by BUCKy (α = 1) from gene trees inferred by MrBayes v3.2.1.

**Figure S3.** The concatenated phylogenies generated (in RAxML) using **(A)** 72 concatenated chloroplast genes and **(B)** ten concatenated mitochondrial genes. Bootstrap support is indicated above the focal branch.

Literature Cited

Alverson AJ, et al. 2010. Insights into the evolution of mitochondrial genome size from complete sequences of Citrullus lanatus and Cucurbita pepo (Cucurbitaceae). Mol Biol Evol 27:1436-1448.

Campbell MS, et al. 2014. MAKER-P: a tool kit for the rapid creation, management, and quality control of plant genome annotations. Plant Physiol 164:513-524.

Dobin A, et al. 2013. STAR: ultrafast universal RNA-seq aligner. Bioinformatics 29:15-21.

Fontaine MC, et al. 2015. Extensive introgression in a malaria vector species complex revealed by phylogenomics. Science 347:1258524.

Huelsenbeck JP, Ronquist F. 2001. MRBAYES: Bayesian inference of phylogenetic trees. Bioinformatics 17:754-755.

Jones P, et al. 2014. InterProScan 5: genome-scale protein function classification. Bioinformatics 30:1236-1240.

Korf I. 2004. Gene finding in novel genomes. BMC Bioinformatics 5:1.

Lamesch P, et al. 2012. The Arabidopsis Information Resource (TAIR): improved gene annotation and new tools. Nucleic Acids Res 40:D1202-D1210.

Larget BR, Kotha SK, Dewey CN, Ané C. 2010. BUCKy: gene tree/species tree reconciliation with Bayesian concordance analysis. Bioinformatics 26:2910-2911.

Li H, Durbin R. 2010. Fast and accurate long-read alignment with Burrows–Wheeler transform. Bioinformatics 26:589-595.

Li H, et al. 2009. The sequence alignment/map format and SAMtools. Bioinformatics 25:2078-2079.

Liao Y, Smyth GK, Shi W. 2013. featureCounts: an efficient general purpose program for assigning sequence reads to genomic features. Bioinformatics 30:923-930.

Löytynoja A, Goldman N. 2005. An algorithm for progressive multiple alignment of sequences with insertions. Proc Natl Acad Sci U S A 102:10557-10562.

Lukashin AV, Borodovsky M. 1998. GeneMark. hmm: new solutions for gene finding. Nucleic Acids Res 26:1107-1115.

Ma J, Bennetzen JL. 2004. Rapid recent growth and divergence of rice nuclear genomes. Proceedings of the National Academy of Sciences 101:12404-12410.

Magrane M, Consortium U. 2011. UniProt Knowledgebase: a hub of integrated protein data. Database 2011:bar009.

Mirarab S, Warnow T. 2015. ASTRAL-II: coalescent-based species tree estimation with many hundreds of taxa and thousands of genes. Bioinformatics 31:i44-i52.

Okonechnikov K, Conesa A, García-Alcalde F. 2016. Qualimap 2: advanced multi-sample quality control for high-throughput sequencing data. Bioinformatics 32:292-294.

Ou S, Jiang N. 2018. LTR_retriever: A Highly Accurate And Sensitive Program For Identification Of LTR Retrotransposons. Plant Physiol 176:1410-1422.

Parra G, Bradnam K, Korf I. 2007. CEGMA: a pipeline to accurately annotate core genes in eukaryotic genomes. Bioinformatics 23:1061-1067.

Revell LJ. 2012. phytools: an R package for phylogenetic comparative biology (and other things). Methods Ecol Evol 3:217-223.

Salichos L, Rokas A. 2013. Inferring ancient divergences requires genes with strong phylogenetic signals. Nature 497:327-331.

Soorni A, Haak D, Zaitlin D, Bombarely A. 2017. Organelle_PBA, a pipeline for assembling chloroplast and mitochondrial genomes from PacBio DNA sequencing data. BMC Genomics 18:49.

Stamatakis A. 2006. RAxML-VI-HPC: maximum likelihood-based phylogenetic analyses with thousands of taxa and mixed models. Bioinformatics 22:2688-2690.

Stanke M, et al. 2006. AUGUSTUS: ab initio prediction of alternative transcripts. Nucleic Acids Res 34:W435-W439.

The Tomato Genome Consortium. 2012. The tomato genome sequence provides insights into fleshy fruit evolution. Nature 485:635-641.

Wu M, Kostyun JL, Hahn MW, Moyle L. 2018. Dissecting the basis of novel trait evolution in a radiation with widespread phylogenetic discordance. Mol Ecol 27:3301-3316.

Wyman SK, Jansen RK, Boore JL. 2004. Automatic annotation of organellar genomes with DOGMA. Bioinformatics 20:3252-3255.
